# Correction: Xu et al. Hyaluronic Acid Interacting Molecules Mediated Crosstalk between Cancer Cells and Microenvironment from Primary Tumour to Distant Metastasis. *Cancers* 2024, *16*, 1907

**DOI:** 10.3390/cancers17030549

**Published:** 2025-02-06

**Authors:** Yali Xu, Johannes Benedikt, Lin Ye

**Affiliations:** 1 Cardiff China Medical Research Collaborative, Division of Cancer and Genetics, Cardiff University School of Medicine, Cardiff CF14 4XN, UK; xuy109@cardiff.ac.uk; 2 School of Engineering, Cardiff University, Cardiff CF24 3AA, UK; benedikt@cardiff.ac.uk

In the original publication [1], there was a mistake due to a technical issue during the conversion of the authors’ galley proof—Correct Figures 1–3 are missing. Figure 1 is misplaced with the correct Figure 4. Also, Figures 2–4 are duplicated figures of Figures 5–7. The corrected Figure 1, Figure 2, Figure 3, Figure 4, Figure 5, Figure 6 and Figure 7 appear below. The scientific conclusions are unaffected. This correction was approved by the Academic Editor. The original publication has also been updated.

## Figures and Tables

**Figure 1:**
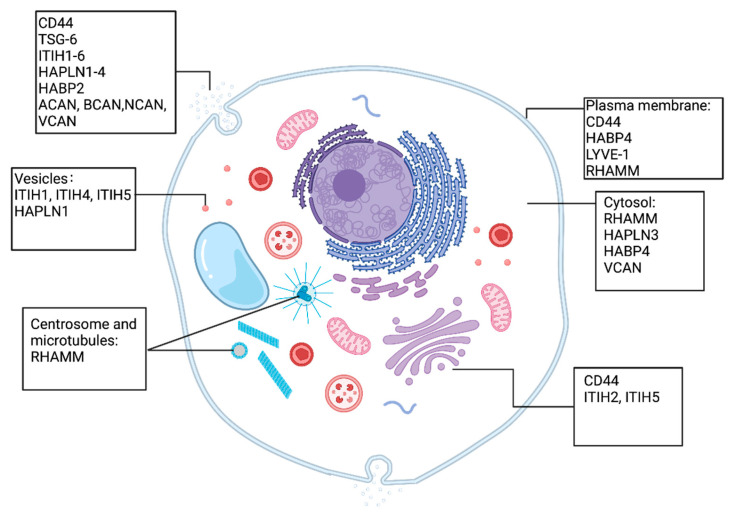
Subcellular location of HAIMs. Differences in the distribution of the HAIMs indicate a wide range of biological functions affected by these molecules. The figure was created with BioRender (www.Biorender.com, accessed on 8 May 2024).

**Figure 2:**
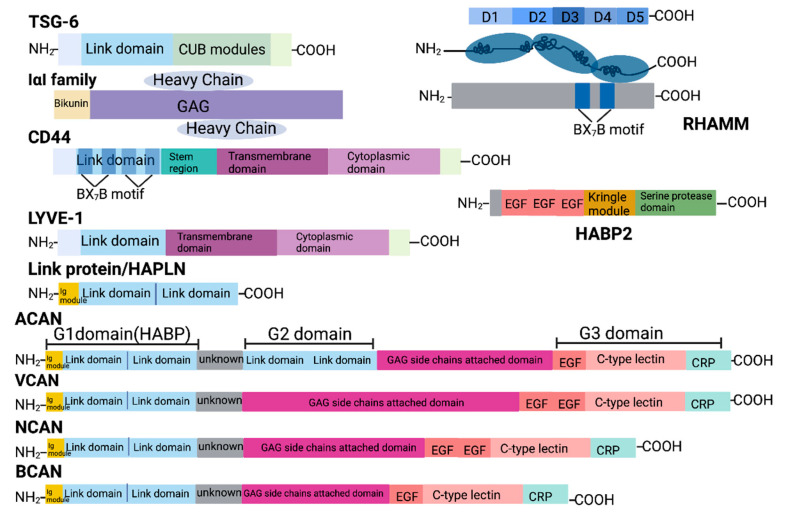
Protein structure of HAIMs. Domain architecture of HAIMs. The link domain on the N-terminal of TSG-6, CD44, LYVE-1, HAPLNs and lecticans is the HA-binding domain, while in RHAMM, BX7B motif helps to bind HA. The G1 domain in the lecticans is responsible for HA binding; the G3 domain binds to ECM molecules like Tenascin and carbohydrate, including GAGs on the cell membrane. G2 domain is only found in ACAN, but its function remains unknown. CUB = C1r/C1s, Uegf, Bmp1; Within the BX7B motif, B = arginine (R) or lysine (K); X = non-acidic amino; GAG = glycosaminoglycans; EGF = epidermal growth factor (EGF)-like motif; CRP = complement regulatory protein repeat. This figure was created with www.BioRender.com (accessed on 8 May 2024).

**Figure 3:**
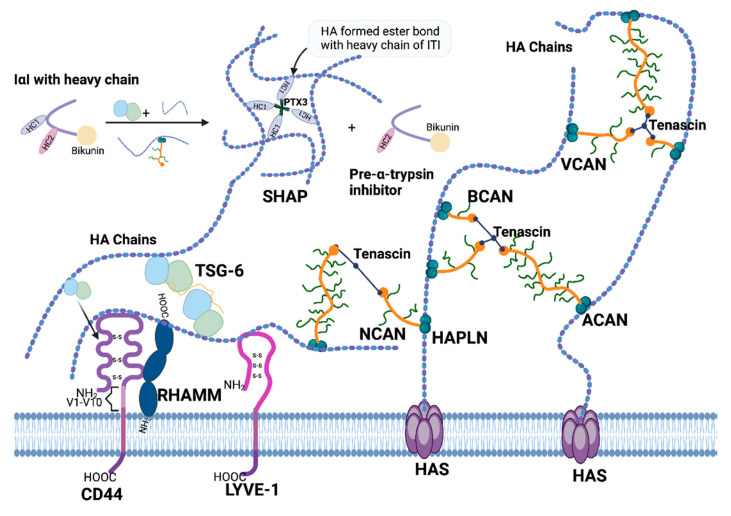
Interactions between HAIMs and HA in the ECM. HAIMs interact with HA to organise ECM. HA is produced directly by HAS. TSG-6 and VCAN assist with the transport of HC from IαI family members to HA chains. HCs linked to HA chains via ester bond and Pre-α-trypsin inhibitor are released. PTX3 helps with further HA organisation in the mammalian oocytes complex matrix. TSG-6 itself interacts with CD44 and enables CD44 to form a complex with HA. The induction of dimerization in TSG-6 by HA leads to the crosslinking of the HA polysaccharide. HAPLNs binding on the G1 domain of lecticans and tenascin’s binding to lecticans on their G3 domain also help to construct HA scaffold. PTX3 = Pentraxin-3; HC = heavy chain. This figure was created with BioRender (www.Biorender.com, accessed on 8 May 2024).

**Figure 4:**
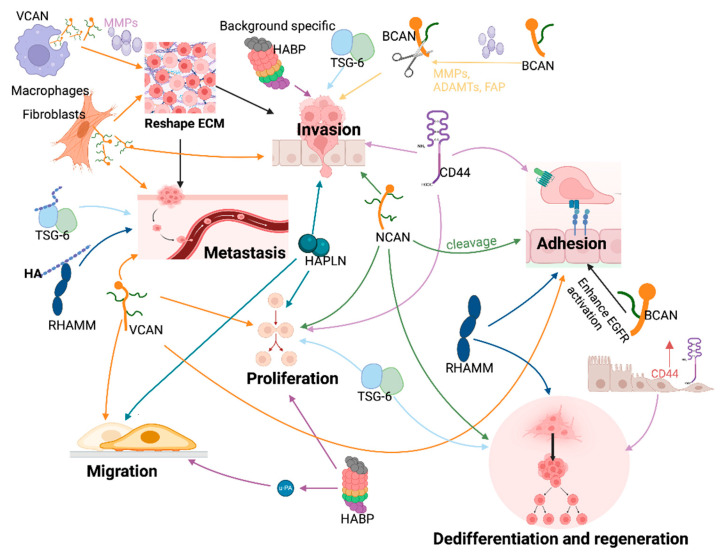
HAIMs modulate cellular functions and the dissemination of cancer cells by interacting with ECM components. HAIMs from cancer cells, immune cells and mesenchymal cells all contributed to the abnormal HAIM levels in tumours. These HAIMs either function alone or form complexes with other HAIMs and ECM components including HA to alter malignant cellular behaviours. Cleavage of lecticans will affect their function. Various colours are used for indicating different molecules. Arrows are used to show their promoting effects. MMP = matrix metalloproteinase; ADAMTS = A disintegrin and metalloproteinase with thrombospondin motifs; FAP = fibroblast activation protein; EGFR = epidermal growth factor receptor. The figure was prepared with BioRender (www.Biorender.com, accessed on 8 May 2024).

**Figure 5:**
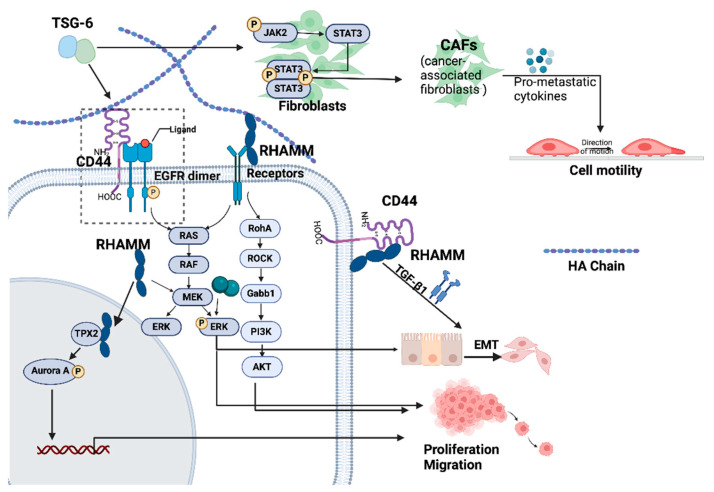
The molecular machinery of CD44, TSG-6, HAPLN and RHAMM regulated tumour proliferation, movement and metastasis. TSG-6 can facilitate CD44-EGFR interaction. HA/CD44 complex enhances EGFR-promoted cell proliferation and EMT via the RAS/TAF/MEK/ERK pathway. Membrane-anchored RHAMM–HA interaction activates RHO/ROCK pathway and therefore promoted cell migration and proliferation via unknown transmembrane receptors. Intercellular RHAMM enter the nucleus and binds to and stabilizes TPX2 to activate AURKA, leading to enhanced proliferation and migration. TSG-6 converts normal fibroblasts to CAF and therefore promotes CRC metastasis. RHAMM/CD44 complex also promotes EMT with the involvement of TGF-β1. AUKA = Aurora kinase A; TGFβ = transforming growth factor beta; EMT = epithelial–mesenchymal transition; EGFR = epidermal growth factor receptor. Created with BioRender (www.Biorender.com, accessed on 8 May 2024).

**Figure 6:**
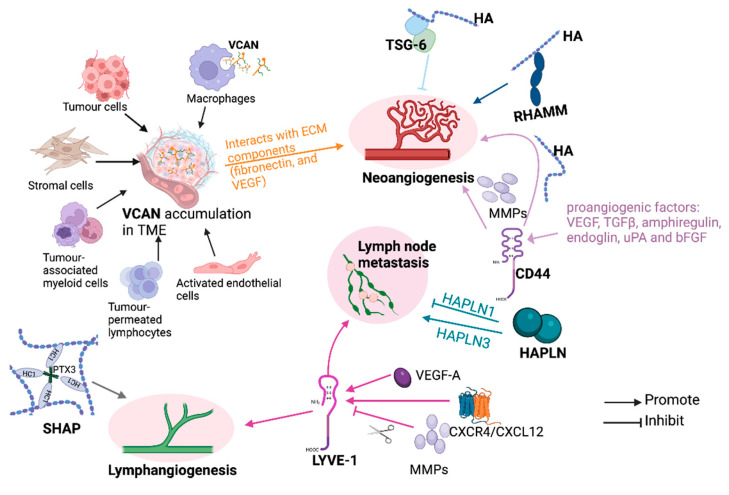
HAIMs’ involvement in angiogenesis and lymphangiogenesis. VCAN, TSG-6, RHAMM and CD44 contribute to angiogenesis while SHAP, LYVE-1 and HAPLNs are involved in lymph angiogenesis and lymph node metastasis. Various colours are used for the purpose of grouping, indicating distinct molecular functions. VEGF = vascular endothelial growth factor; TGFβ = transforming growth factor beta; uPA = urokinase-type plasminogen activator; bFGF = basic fibroblast growth factor; CXCR4/CXCL12 = chemokine (C-X-C motif) receptor 4/chemokine (C-X-C motif) ligand 12. Created with BioRender (www.Biorender.com, accessed on 8 May 2024).

**Figure 7:**
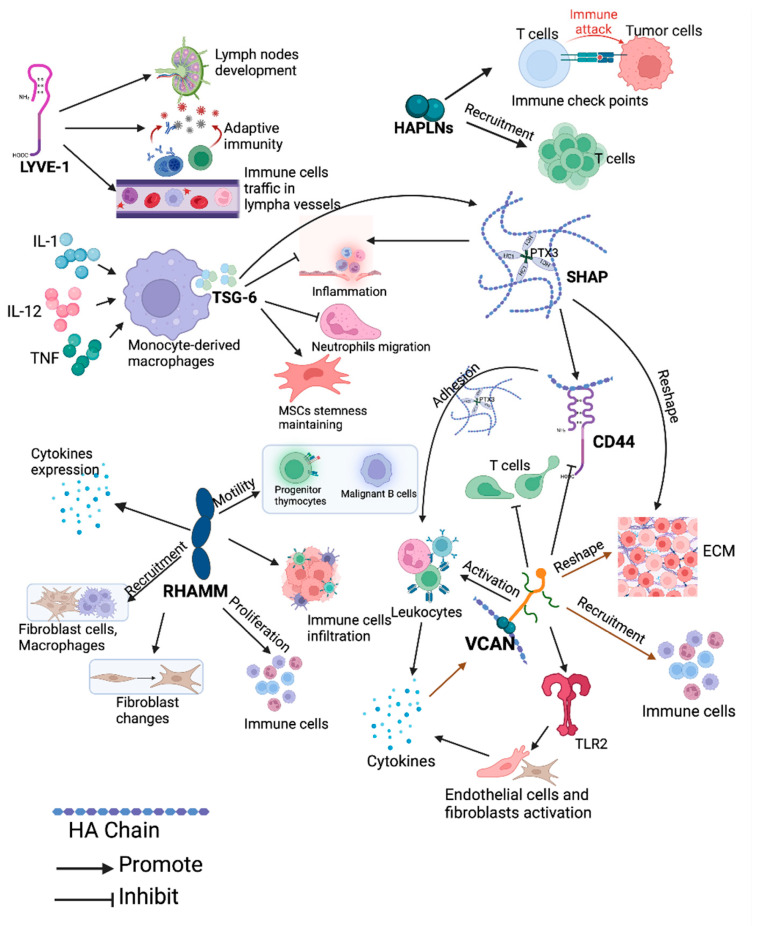
HAIMs’ involvement in immune regulation. Various colours are used for the purpose of grouping, indicating distinct molecular functions. HC = heavy chain; PTX-3 = pentraxin-3; IL = interleukin; TNF = tumour necrosis factor; TLR = toll-like receptor. Created with BioRender (www.Biorender.com, accessed on 8 May 2024).
